# Is Jaw in a Day® reconstruction feasible in patients undergoing robotic neck dissection for oral cancer?: a case series with comparative analysis

**DOI:** 10.1186/s40902-025-00489-2

**Published:** 2025-10-16

**Authors:** Daniel Wilfredo Banegas, Jung Min Cho, Mi-Kyung Gong, Hyounmin Kim, Woong Nam, Hyung Jun Kim, Yoon Woo Koh, Dongwook Kim

**Affiliations:** 1 Maxillofacial Surgery Department, National Medical Center “20 de Noviembre” ISSSTE, Ciudad de México, Mexico; 2 Department of Oral and Maxillofacial Surgery, Yonsei University College of Dentistry, Seoul, Korea, Republic of; 3 Department of Otorhinolaryngology, Yonsei University College of Medicine, Seoul, Korea, Republic of; 4 Oral Science Research Center, Yonsei University College of Dentistry, Seoul, Korea, Republic of

**Keywords:** Robotic surgical procedures, Neck dissection, Oral neoplasms, Mandibular reconstruction, Dental implants, 3D printing

## Abstract

**Background:**

Immediate dental implant placement with implant-supported prostheses enables single-stage functional and aesthetic rehabilitation during jaw reconstruction, a technique referred to as “Jaw in a Day®” (JIAD). This study evaluated the feasibility of the JIAD technique combined with retroauricular robot-assisted neck dissection (RA-RAND) in patients with oral cancer undergoing mandibular reconstruction.

**Materials and methods:**

We retrospectively reviewed 75 patients who underwent mandibular reconstruction using fibula free flaps from September 2020 to February 2024. Among them, 31 patients were eligible for analysis. Seven patients had retroauricular robot-assisted neck dissection (RA-RAND), and 24 patients had conventional transcervical neck dissection (CTND). Two patients in the RA-RAND group and nine in the CTND group underwent the JIAD procedure. We compared the time from data acquisition to surgery, reconstruction time, total operation time, and length of hospital stay between the groups.

**Results:**

No significant differences were observed between the RA-RAND and CTND groups in terms of reconstruction time (median 431 min, IQR 274–442 vs. 310 min, IQR 236–420; *p* = 0.435) or hospital stay (median 20 days, IQR 17–22 vs. 20 days, IQR 18–33; *p* = 0.275), although the total operation time was significantly longer in the RA-RAND group (median 831 min, IQR 702–898 vs. 526 min, IQR 444–615; *p* = 0.002). Within the RA-RAND cohort, there were no significant differences between the JIAD and non-JIAD groups regarding time from data acquisition to surgery (median 17.5 days, IQR 14.2–20.8 vs. 13.0 days, IQR 8.0–24.0; *p* = 1.000), reconstruction time (median 352.5 min, IQR 311.2–393.8 vs. 431.0 min, IQR 278.0–450.0; *p* = 0.857), total operation time (median 863.5 min, IQR 847.2–879.8 vs. 701.5 min, IQR 649.0–751.5; *p* = 0.857), or length of hospital stay (median 18.5 days, IQR 15.2–21.8 vs. 20.0 days, IQR 18.0–22.0; *p* = 0.762).

**Conclusion:**

Based on this case series and comparative analysis, the combination of the JIAD technique with RA-RAND appears technically feasible and does not prolong the operative or postoperative course. However, larger studies are required to confirm these findings.

## Introduction

Retroauricular robot-assisted neck dissection (RA-RAND) is used in the surgical management of oral cancer, offering favorable cosmetic outcomes by avoiding visible neck scars [1]. Previous studies have shown that free flap reconstruction can be successfully performed through this approach, not only for soft tissue but also for bony reconstruction of the mandible, with outcomes comparable to those of conventional transcervical approaches [2].

A recent trend in mandibular reconstruction involves the placement of dental implants and the immediate delivery of a provisional prosthesis at the time of fibula free flap (FFF) reconstruction—a technique commonly known as the “Jaw in a Day®” (JIAD) [3]. The JIAD technique was registered and utilized as a trademark by David Hirsch, Jamie Levine, and Lawrence Brecht [4]. This JIAD approach has demonstrated particular advantages in oral cancer patients by increasing implant survival and helping to avoid osteoradionecrosis, a devastating complication of delayed post-radiation implant placement [5].

This study aims to evaluate the feasibility of JIAD in patients undergoing RA-RAND. Specifically, we investigated whether simultaneous implant placement and prosthesis delivery can be performed without delaying surgery or increasing operative time compared to conventional reconstruction. The digital workflow is also described.

## Materials and methods

### Patients

**Fig. 1:**
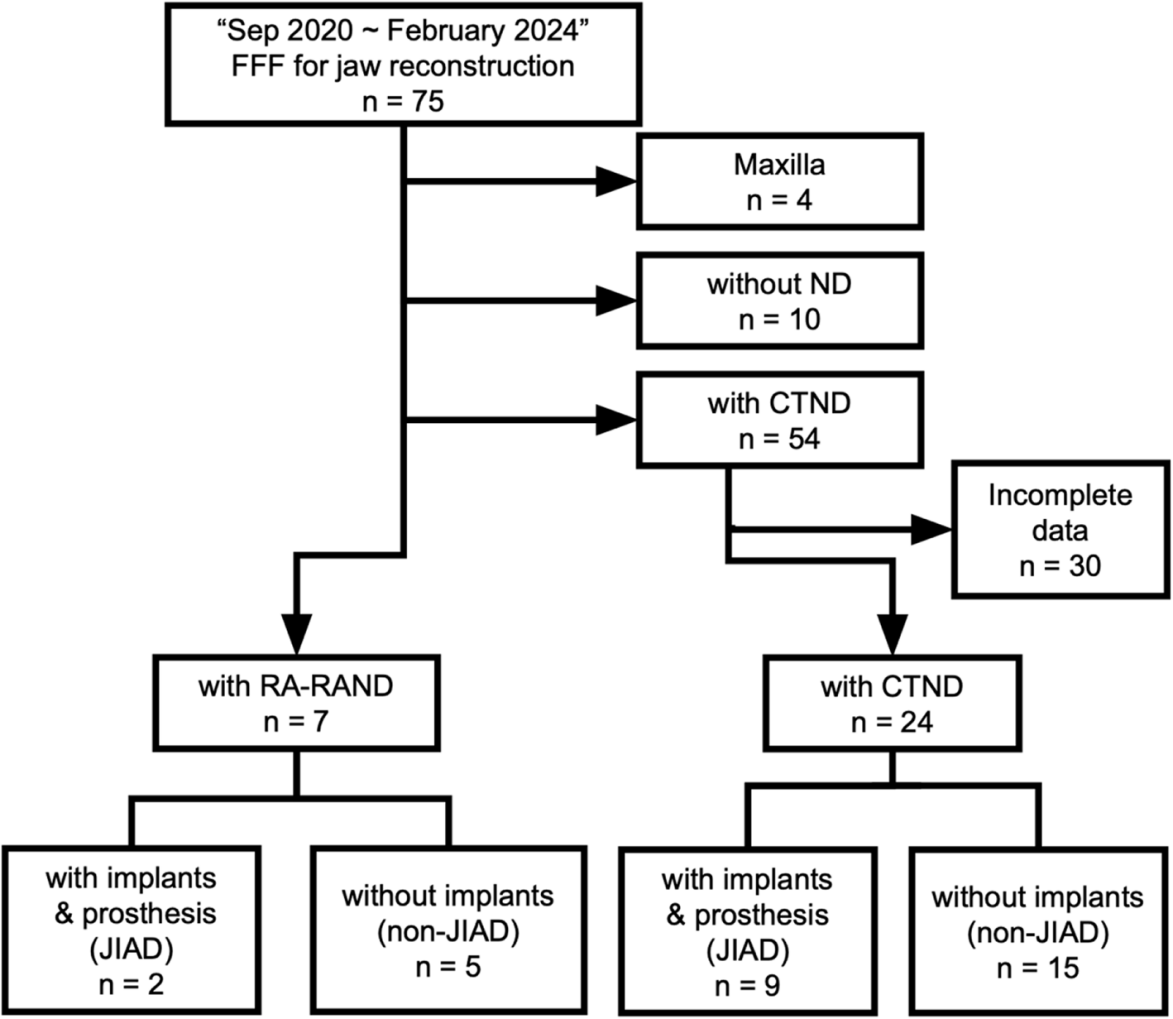
Flow diagram showing patient screening, exclusion, and inclusion. FFF: fibula free flap, ND: neck dissection, CTND: conventional transcervical neck dissection, RA-RAND: retroauricular robot-assisted neck dissection, JIAD: Jaw in a Day

We retrospectively reviewed 75 consecutive patients who underwent jaw reconstruction with FFF by a single reconstructive surgeon between September 2020 and February 2024. After excluding 4 maxillary reconstruction cases, 10 without neck dissections (ND), and 30 CTND cases with incomplete data, a total of 31 patients who underwent ND and mandibular reconstruction with FFF were included for analysis. Two patients in the RA-RAND group and nine in the CTND group underwent the JIAD procedure (Fig. 1).

Clinical and demographic data, including patient age, gender, Brown classification of mandibular defects, surgical approach (two-team vs. sequential), flap survival, and perioperative timing parameters, were retrospectively collected from electronic medical records. Days from data acquisition to surgery, reconstruction time, total operation time, and length of hospital stay were also retrospectively collected from medical records. The reconstruction time was defined as the period from the initial incision at the donor site to the completion of all reconstruction-related procedures, including implant placement, prosthesis delivery, flap insetting, vascular anastomosis, donor site repair, and skin suturing. This retrospective review was approved by the institutional review board (IRB).

### Digital workflow

#### Digital data acquisition

The images below were obtained before planning for the JIAD surgery. CT data will be in Digital Imaging and Communications in Medicine (DICOM) format, and the patient’s dental cast will be in a Stereolithography (STL) file format.

1. Computed tomography (CT) of the maxillofacial skeleton.
2. CT angiography of bilateral lower extremities: This data will provide information related to the fibula and the presence of an anomaly in the peroneal vessel [6].
3. STL file of pre-surgical dentition: This is required for immediate prosthesis planning and can be obtained directly with an intraoral scanner or indirectly with a desktop 3D scanner by scanning the cast from a dental impression. In this digital workflow, the scanning process is carried out using a Creality 3D scanner (ShenZhen Creality 3D Technology Co., Ltd, Shenzhen, China), a desktop scanner, by scanning a patient’s dental stone cast. This low-cost scanner offers sufficient quality for provisional immediate prostheses.

#### Virtual surgical planning (VSP) of mandibulectomy and creating a neomandible

**Fig. 2:**
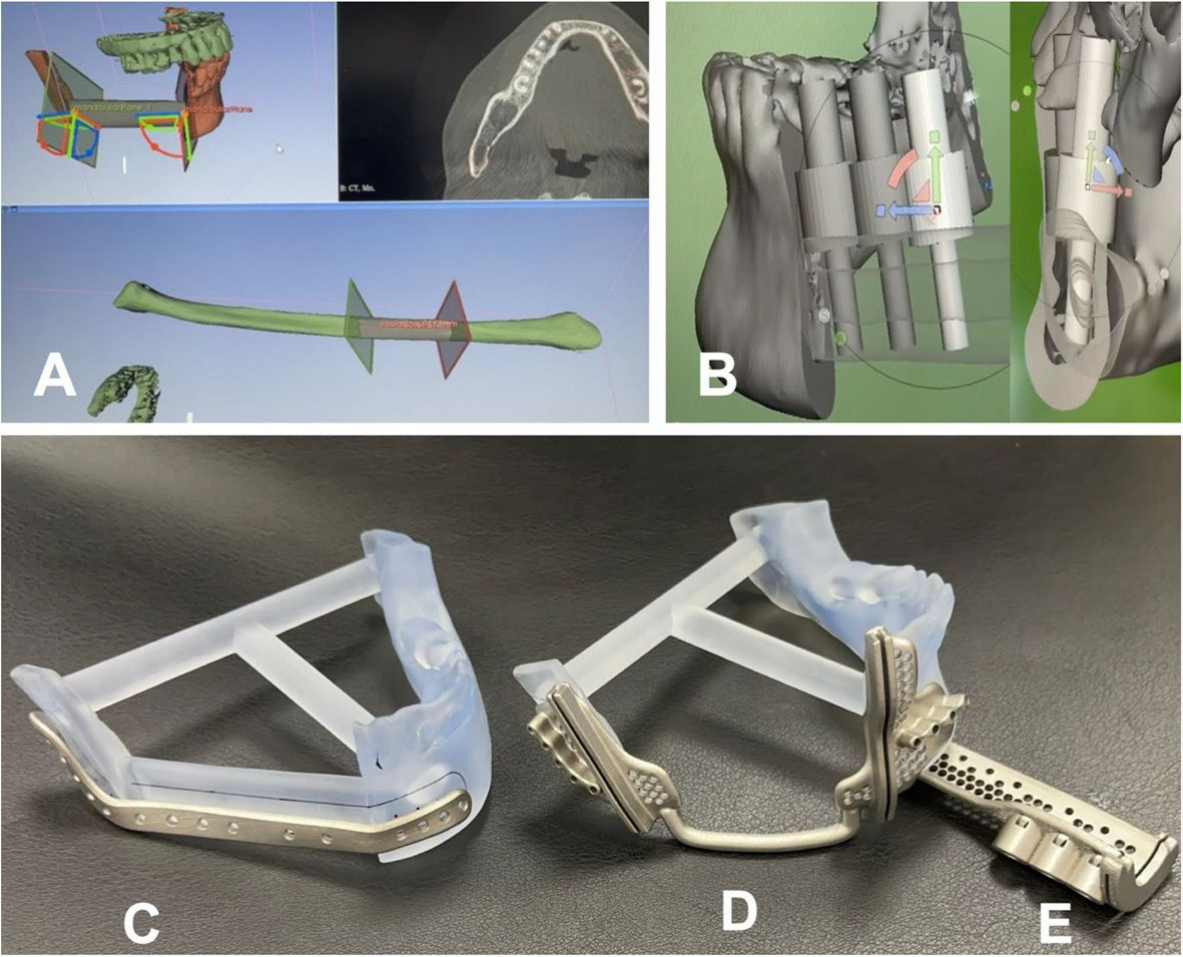
Virtual surgical planning and 3D-printed patient-specific guides and plates. **A** 3D Slicer with the Bone Reconstruction Planner extension for resection and reconstruction planning. **B** Meshmixer for implant planning and prosthesis planning. Making the fibula segments semi-translucent in Meshmixer allows easy visualization and adjustment of implant positions to ensure sufficient surrounding bone within the fibula. The cylindrical structure in the middle of the template serves as a drill guide for implant placement. **C** Customized plate. **D** Mandible cutting guide. **E** Fibula-cutting guide with implant drill guide

Virtual surgical planning (VSP) was performed using 3D Slicer (version 5.8.1; www.slicer.org), a free open-source software with the Bone Reconstruction Planner (BRP) extension [7–9] (Fig. 2A). This enables the creation of 3D segmented models of the reconstructed virtual jaw, referred to as the “*neomandible*.” The resection margin is set where the saw will cut through the middle of a tooth, and not between them, to preserve adequate alveolar bone for the remaining teeth.

The fibula segment is positioned above the inferior border of the mandible in the anterior region and remains low at the proximal region. This enables proper interocclusal distance without compromising aesthetics. The fibula is oriented to place implants at the anterior surface of the fibula, and the lateral surface of the fibula is oriented buccally. This orientation allows the skin paddle to rise from the inferior border to the buccal or labial aspect, resulting in a higher alveolar ridge and a deeper vestibule.

**Fig. 3:**
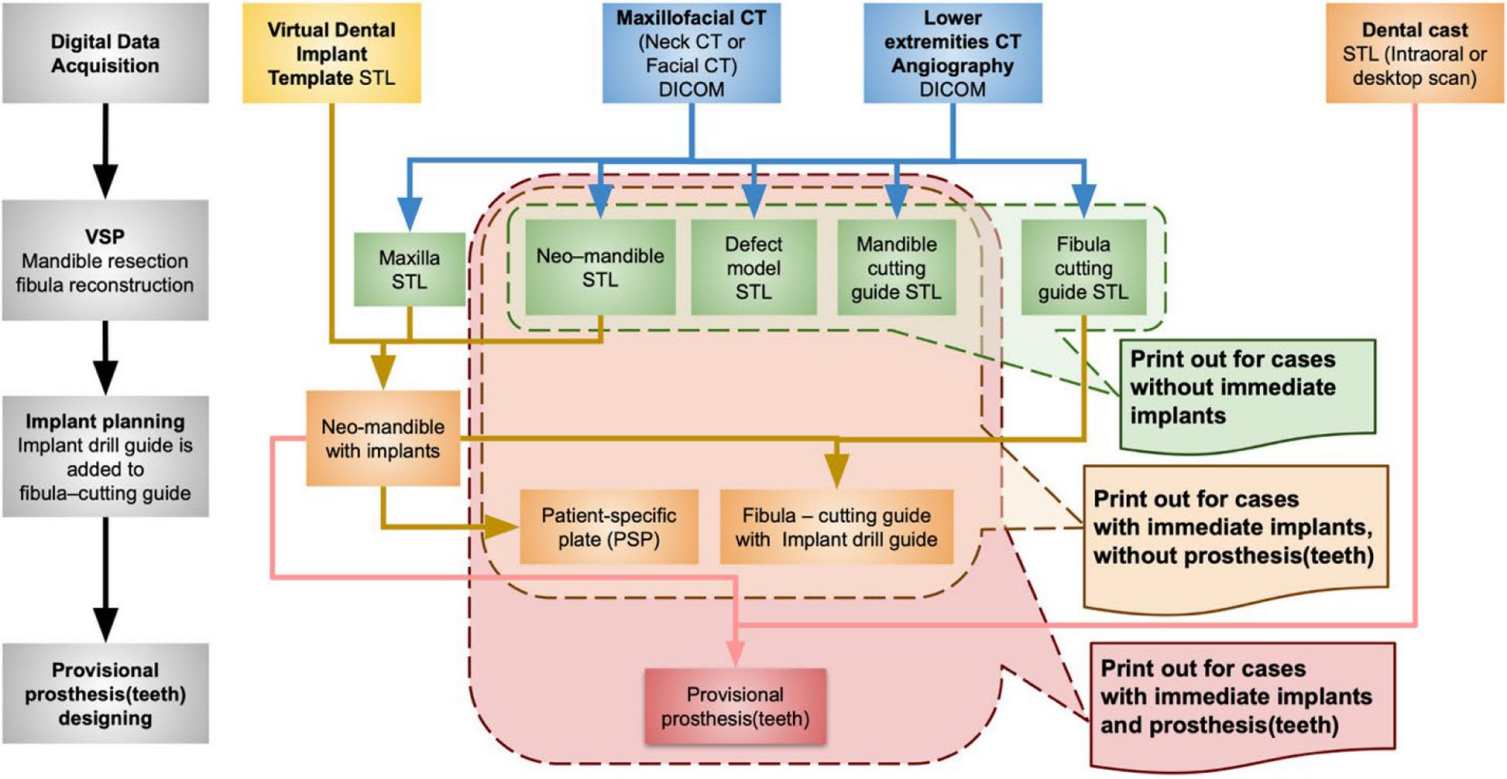
Flowchart of data acquisition, virtual surgical planning, and 3D printing. The STL files can be printed as 3D models at each step according to the reconstruction goal: without immediate implants, with immediate implants, and with immediate implants and prosthesis

The fibula and mandible cutting guide STLs can be automatically created using the BRP extension in a 3D slicer and printed in-house or sent to 3D printing manufacturers for cases without immediate dental implants (Fig. 3).

#### Planning dental implants on the neomandible

Meshmixer (version 3.5; Autodesk Inc., San Rafael, CA, USA), a free-use software for creating and manipulating 3D files, is used for dental implant planning and placement, as well as for contouring, manipulation, and preparing the 3D-printed dental prosthesis. The *neomandible* exported as an STL is imported into Meshmixer. A virtual dental implant template has been created for the OneGuide® system (Osstem Implant Co., Seoul, Korea), which includes a 4.0 × 10.0 mm fixture template, drill guide, and a column for the access hole, all combined into a single STL file [10] (Fig. 2B).

As for the implant position along the fibula segments, the implant must be at least 3 mm away from the fibula osteotomy edges [11]. The parallelism between implants is preferred, but not mandatory. Once the implant’s final position has been determined, these can be exported as new STL files (Fig. 3).

For cases involving immediate dental implants, the *neomandible* and dental implants STL are sent to the custom plate and surgical guide manufacturer (Cubelabs Inc., Gangneung, Kangwon-do, Korea). The mandible cutting guides are connected into a single unit for accuracy (Fig. 2C-E). The fibula cutting guide contains an implant drill guide. Both guides contain drill holes for the fixation of custom plates, thereby enabling the maintenance and restoration of occlusion without additional fixation devices.

### Intra-operative phase

#### Robot-assisted neck dissection and mandibulectomy

**Fig. 4:**
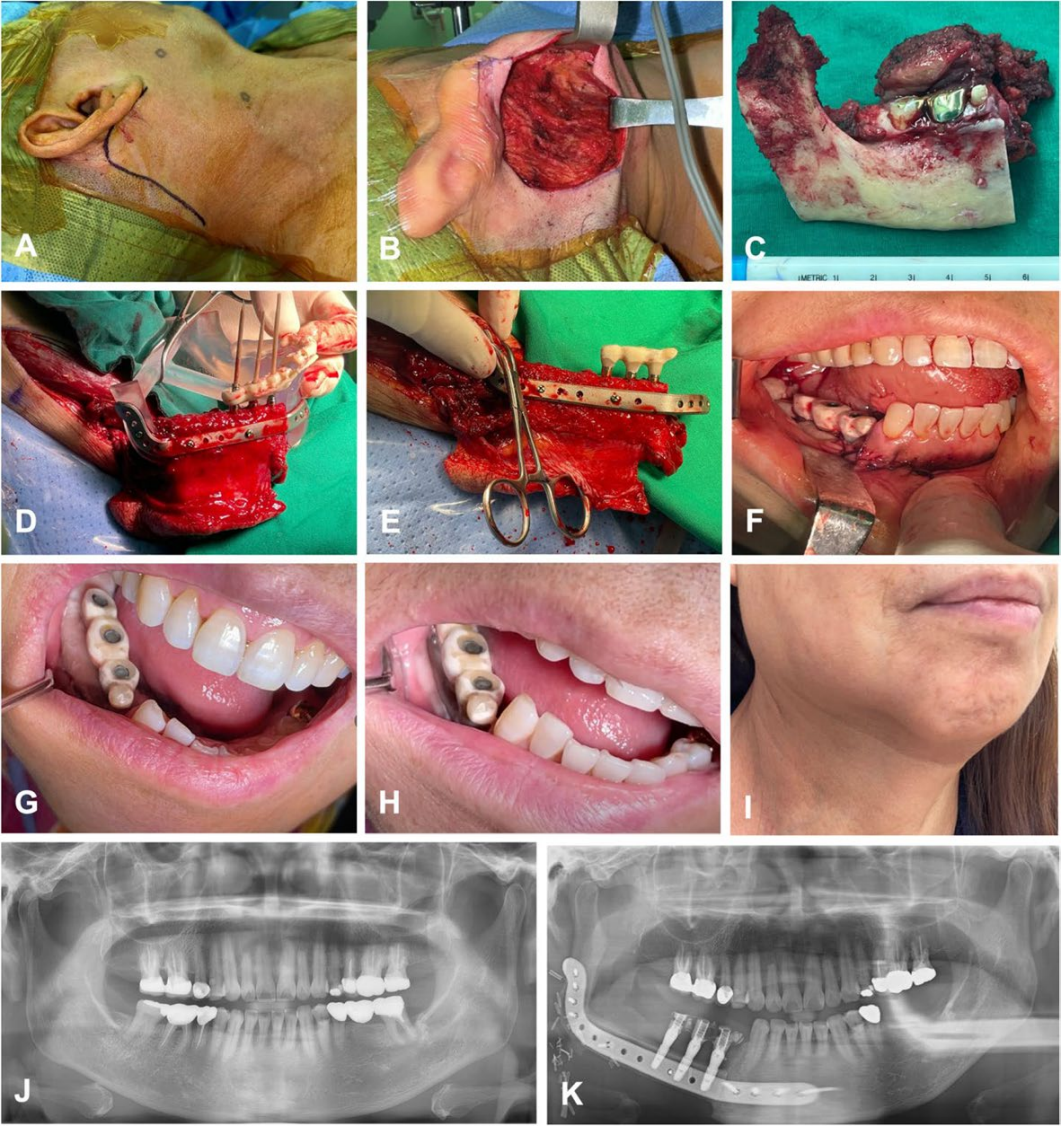
RA-RAND, mandibulectomy, and JIAD procedure #2. **A**, **B** Retroauricular incision and subplatysmal flap elevation. **C** Mandibulectomy specimen. **D** The fibula segment is fixated to the defect model using the PSP, and the provisional prosthesis is delivered while the vascular pedicle is still being maintained at the leg. **E** A provisional prosthesis is placed after trimming, polishing, and disinfection. **F** Immediate postoperative intraoral photo. **G** Intraoral view at 1 month postoperatively. **H** Intraoral view at 5 months postoperatively showing the skin paddle naturally contracted and resembling attached gingiva, without any additional soft tissue procedure. The patient also had postoperative radiation therapy. **I** Five-month postoperative view demonstrating no apparent scarring and good preservation of the preoperative mandibular contour. **J** Preoperative panoramic radiograph. **K** Postoperative panoramic radiograph

**Fig. 5:**
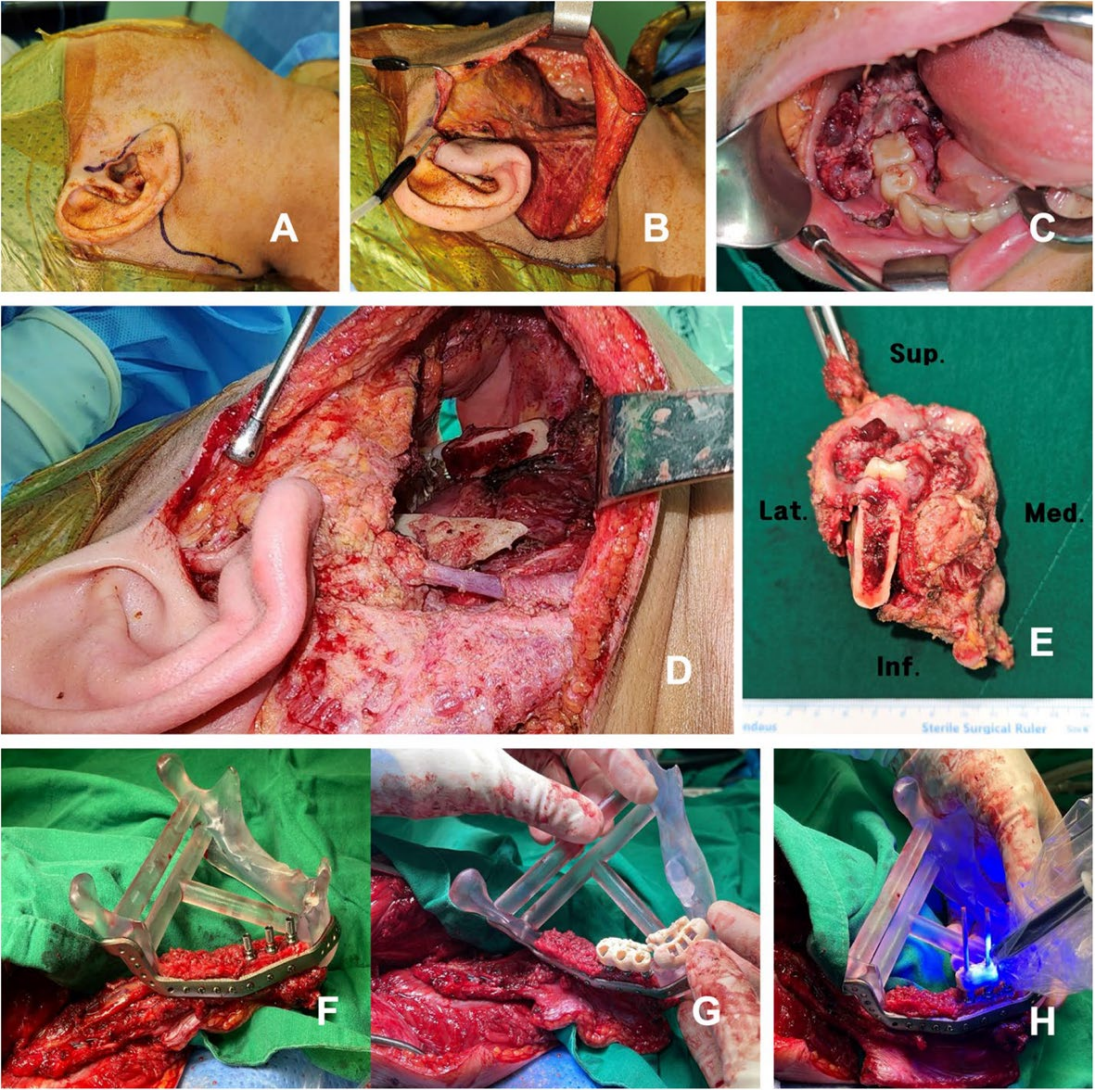
RA-RAND, mandibulectomy, and JIAD procedure in another case. **A** Retroauricular incision with preauricular extension. **B** Subplatysmal flap elevation and retractor application. **C** Intraoral view of the tumor. **D** Defect after mandibulectomy showing the proximal portion of the mandible can be adequately accessed through the RA approach. **E** Mandibulectomy specimen. **F** FFF with implants contoured and fixated to the PSP, mounted to the defect model. **G** Provisional prosthesis try-in. **H** Relining the provisional prosthesis

RA-RAND was performed with the Da Vinci® Surgical System (Intuitive Surgical Inc., Sunnyvale, CA, USA). The sternocleidomastoid muscle (SCM), internal jugular vein (IJV), and spinal accessory nerve (SAN) were identified and preserved. Mandibulectomy was performed using a cutting guide via a combined intraoral and retroauricular (RA) approach. The distal portion of the mandible was first resected intraorally, followed by proximal resection through the RA approach, which provides adequate access to the mandibular ramus and condyle (Figs. 4A-C, 5A-E).

#### Fibula free flap reconstruction with simultaneous dental implants

The FFF was harvested conventionally, and osteotomies at both the distal and proximal ends were performed. The fibula cutting guide was secured to the fibula, and dental implants were placed using the implant guide integrated into the cutting guide. Osteotomy of the fibula was then performed. The osteotomized fibula segments were fixed to the patient-specific plate (PSP), which was then secured to the defect model. The provisional prosthesis was then relined with light-cured resin. All procedures were performed while maintaining vascular flow before ligating the peroneal artery (Figs. 4D, E, 5F–H).

Following ligation of the peroneal vessels, the flap was transferred to the defect site and inset, with confirmation of sufficient range of motion, balanced movement of the bilateral mandibular condyles, and favorable occlusion. Microanastomosis was then performed, and reconstruction was completed by placing a drain, closing the skin, and repairing the donor site (Fig. 4F–K).

### Statistical analysis

Continuous variables are presented as medians and interquartile ranges (IQRs), and categorical variables as frequencies and percentages. Differences between groups were assessed using the Mann–Whitney *U* test for continuous variables and Fisher’s exact test for categorical variables. A *p* value less than 0.05 was considered statistically significant. All statistical analyses were performed using R software (version 4.5.1; R Foundation for Statistical Computing, Vienna, Austria).

## Results

A comparison between the RA-RAND and the CTND groups revealed no significant difference in terms of gender, age, brown class, and flap survival in both groups (Table 1). There was no significant difference in reconstruction time between the groups (RA-RAND: median 431 min [IQR 274–442] vs. CTND: median 310 min [IQR 236–420], *p* = 0.435). However, the total operation time was significantly longer in the RA-RAND group (median 831 min [IQR 702–898] vs. CTND: median 526 min [IQR 444–615], *p* = 0.002). This may be explained by the fact that the two-team approach was less frequently employed in the RA-RAND group (28.6% vs. 100%, *p* < 0.001), resulting in a longer overall procedure time, as reconstruction followed resection sequentially, despite similar reconstruction times. There was also no significant difference in hospital stay between the two groups (RA-RAND: median 20 days [IQR 17–22] vs. CTND: median 20 days [IQR 18–33], *p* = 0.275) (Table 1).

**Table 1:** Demographic and clinical characteristics of RA-RAND and CT groups

	RA-RAND (*n* = 7)	CTND (*n* = 24)	*p* value^a^
Gender			1.000
Female	3 (42.9%)	10 (41.7%)	
Male	4 (57.1%)	14 (58.3%)	
Age	60 (57–64)	69 (65–76)	0.088
Two-team approach			<0.001
Yes	2 (28.6%)	24 (100.0%)	
No	5 (71.4%)	0 (0.0%)	
Brown class			0.896
I	5 (71.4%)	19 (79.2%)	
II	1 (14.3%)	4 (16.7%)	
III	1 (14.3%)	1 (4.2%)	
Flap survival			-
Yes	7 (100.0%)	24 (100.0%)	
No	0 (0.0%)	0 (0.0%)	
Data acquisition to operation, days	13 (10–24)	10 (7–16)	0.284
Reconstruction time, minutes	431 (274–442)	310 (236–420)	0.435
Total operation time, minutes	831 (702–898)	526 (444–615)	0.002
Hospital stay, days	20 (17–22)	20 (18–33)	0.275

Values are presented as numbers (%) for categorical variables and as medians (interquartile range, IQR) for continuous variables

^a^*p*-values were calculated using Fisher’s exact test for categorical variables and the Mann–Whitney *U* test for continuous variables

When comparing the JIAD and non-JIAD groups within the RA-RAND cohort, there was no significant difference in the time from data acquisition to operation between the JIAD group (median 17.5 days, IQR 14–21) and the non-JIAD group (median 13.0 days, IQR 8–24) (*p* = 1.000). There were also no significant differences in reconstruction time (median 352.5 min, IQR 311–394 for JIAD vs. median 431.0 min, IQR 278–450 for non-JIAD; *p* = 0.857), total operation time (median 863.5 min, IQR 847–880 for JIAD vs. median 702.0 min, IQR 701–900 for non-JIAD; *p* = 0.857), or hospital stay (median 18.5 days, IQR 15–22 for JIAD vs. median 20.0 days, IQR 18–22 for non-JIAD; *p* = 0.762) between the two groups (Table 2).

**Table 2:** Demographic and clinical characteristics of JIAD and non-JIAD patients among the RA-RAND group

Variable	JIAD (*n* = 2)	Non-JIAD (*n* = 5)	*p* value^a^
Gender			0.277
Female	2 (100.0%)	1 (20.0%)	
Male	0 (0.0%)	4 (80.0%)	
Age	52.0 (43.2–60.8)	61.0 (55.0–68.0)	0.298
Brown class			1.000
I	2 (100.0%)	4 (80.0%)	
II	0 (0.0%)	1 (20.0%)	
Data acquisition to operation, days	17.5 (14.2–20.8)	13.0 (8.0–24.0)	1.000
Reconstruction time, minutes	352.5 (311.2–393.8)	431.0 (278.0–450.0)	0.857
Total operation time, minutes	863.5 (847.2–879.8)	701.5 (649.0–751.5)	0.857
Hospital stay, days	18.5 (15.2–21.8)	20.0 (18.0–22.0)	0.762

Values are presented as numbers (%) for categorical variables and as medians (interquartile range, IQR) for continuous variables

^a^*p*-values were calculated using Fisher’s exact test for categorical variables and the Mann–Whitney *U* test for continuous variables

## Discussion

Treating cancer has advanced considerably over the past several decades. In medical oncology, targeted therapy and immunotherapy have brought significant improvements in clinical outcomes. In surgical oncology, robotic surgery has led to many positive changes [12, 13]. In the management of oropharyngeal cancer, the introduction of transoral robotic surgery (TORS) has enabled tumor access without the need for mandibulotomy, thereby improving both functional and aesthetic outcomes [14]. Furthermore, RA-RAND allows for cervical lymph node dissection without leaving a visible neck scar [1]. Furthermore, it is reported that free flap reconstruction following RA-RAND can also be successfully performed [2].

In the field of maxillofacial reconstruction following tumor resection, the introduction of VSP and 3D printing technologies has also brought about substantial changes. It enabled the fabrication of surgical guides, models that can be applied intraoperatively, and patient-specific plates (PSPs), allowing for enhanced precision [15]. The application of these technologies has enabled dental implants to be positioned more precisely in accordance with the surgical plan [16, 17]. The culmination of these technological advances is best represented by the JIAD technique, an innovative procedure in which mandibular reconstruction with an osteocutaneous free flap, implant placement, and prosthetic restoration are all performed in a single operation [3, 4]. Without advanced technologies, accurately placing implants to support properly occluding prostheses is difficult due to potential errors in both jaw reconstruction and implant placement, making these techniques essential [3]. Through VSP, the position and angulation of dental implants in the reconstructed segment can be planned with high precision, and 3D printing technology enables the production of corresponding surgical guides, PSPs, and models for the surgery [18, 19]. Although concerns may arise regarding potential surgical delays due to the time required for virtual planning and 3D printing, the present study demonstrated that, by following the workflow outlined in Fig. 3, surgery can be performed without delay.

The presence of dental implants in mandibles reconstructed with osteocutaneous free flaps significantly enhances functional rehabilitation, as dentures alone provide limited masticatory function [20]. Therefore, performing implant placement during a single reconstructive surgery offers substantial benefits, not only by reducing the number of surgeries but also by improving the success of the implant and mitigating the risk of devastating complications such as osteoradionecrosis. Oral cancer patients often require postoperative radiation therapy, during which irradiated bone becomes avascular, acellular, and hypoxic, markedly increasing the risk of implant failure and subsequent radiation osteonecrosis [21, 22]. The risk remains elevated even if more than 2 years have elapsed after radiation therapy when a critical dose has been delivered to the bone [23]. To avoid such complications, implants must be placed before radiation therapy, and the JIAD procedure enables this prevention [5]. Since adverse changes in the reconstructed bone do not occur immediately after the initiation of radiation therapy, starting radiation treatment on schedule postoperatively still allows sufficient time for osseointegration when implants are placed concurrently with reconstruction [24]. Furthermore, effective strategies for peri-implant soft tissue management have been developed, overcoming challenges that were previously a concern when placing implants in mandibles reconstructed with osteocutaneous free flaps [24]. Recently, nerve reconstruction has also been performed in patients undergoing mandibular reconstruction and has been reported to reduce neuropathic pain and improve patient outcomes significantly [25].

Based on the above considerations, it is evident that combining robotic surgery and the JIAD approach constitutes a beneficial strategy for patients. In this study, no significant differences were observed in reconstruction time between the JIAD group (with implant placement) and the non-JIAD group (without implant placement) among RA-RAND patients undergoing mandibular reconstruction with osteocutaneous free flaps. Previous studies have also shown that free flap reconstruction via RA-RAND does not require more reconstruction time compared to the traditional transcervical approach [2]. The longer overall procedure time observed in the RA-RAND group may be partly explained by the less frequent use of the two-team approach. Importantly, this was not due to technical or spatial limitations of the robotic approach. In the number of cases where RA-RAND was performed as a collaborative surgery, the reconstructive surgeon could join after resection was completed, thereby necessitating a sequential rather than simultaneous workflow.

When considering the costs of delayed implant placement requiring additional surgery, simultaneous implant placement may offer cost advantages. For patients undergoing radiation therapy, delayed implant placement carries an increased risk of complications, which not only affects the individual but also raises societal healthcare costs, further supporting the benefits of concurrent reconstruction and implant placement.

This study has several limitations. First, the sample size for JIAD cases within the RA-RAND group is relatively small (*n* = 2), which limits the statistical power and generalizability of our findings. Second, our analysis primarily focused on surgical timing parameters and did not include patient-reported quality of life measures. Third, the retrospective nature of this study introduces potential selection bias and limits our ability to control for confounding variables.

This study introduces a digital workflow that relies solely on free software programs, all of which are accompanied by easily accessible online tutorials. This cost-effective and user-friendly approach facilitates widespread adoption, providing an opportunity to develop a customized in-house protocol for JIAD using open-source software and affordable equipment, thereby enabling the process to be completed efficiently and on time.

## Conclusion

While social withdrawal caused by scarring and difficulties associated with impaired masticatory function present significant challenges, advances in modern reconstructive techniques and robotic technologies have made it possible to effectively overcome these issues, thereby enabling cancer survivors to lead fulfilling lives. In this context, our case series and comparative analysis, combining the JIAD technique with RA-RAND, appear technically feasible without prolonging the operative or postoperative course, although larger studies are needed to confirm these findings.

## Data Availability

No datasets were generated or analysed during the current study.

## Abbreviations

RA-RAND: Retroauricular robot-assisted neck dissection
JIAD: Jaw in a Day
CTND: Conventional transcervical neck dissection
FFF: Fibula free flap
ND: Neck dissection
VSP: Virtual surgical planning
STL: Stereolithography (file format)
DICOM: Digital Imaging and Communications in Medicine
BRP: Bone Reconstruction Planner
PSP: Patient-specific plate
SCM: Sternocleidomastoid muscle
IJV: Internal jugular vein
SAN: Spinal accessory nerve
IQR: Interquartile range
TORS: Transoral robotic surgery
